# Supplementary material for: Evaluating leishmanicidal effects of Lucilia sericata products in combination with Apis mellifera honey using an in vitro model
Source: PLoS One. 2023 Aug 3;18(8):e0283355. doi: 10.1371/journal.pone.0283355 (PMC10399734; doi:10.1371/journal.pone.0283355)
Supplement: S1 File — (PDF) [file pone.0283355.s001.pdf]

Promastigote	Crude ES + honey 24h	Crude ES + honey 24h	Crude ES + honey 24h
0	70	70	70
25	46/8	44/2	48/6
50	40/6	38	42
100	34/7	34	36
150	25/97	24	25
200	12	11	13
250	5/4	4/5	6
300	0	0	0
350	0	0	0
	Crude ES + honey 48h	Crude ES + honey 48h	Crude ES + honey48h
0	70	70	70
25	39/7	40	38
50	27/2	27	28
100	20/52	18/5	20
150	11/1	10	12/5
200	5/86	4	4/5
250	0	0	0
300	0	0	0
350	0	0	0
	Crude ES + honey72h	Crude ES + honey 72h	Crude ES + honey72h
0	70	70	70
25	28/1	28	26
50	22/95	21	22
100	11/6	11	12
150	7/72	8	7
200	3	2	2/5
250	0	0	0
300	0	0	0
350	0	0	0

Promastigote	Crude ES + honey 24h	Crude ES + honey 24h	Crude ES + honey 24h
0	70	70	70
25	53/5	52/5	55
50	45	42	48
100	38/4	37	40
150	29/5	28	30
200	22/2	21	23/5
250	15/2	14	16
300	6/72	5	7
350	0	0	0
	ES >10 kDa + honey 48h	ES >10 kDa + honey 48h	ES >10 kDa + honey 48h
0	70	70	70
25	43	42	45
50	38/1	39	37/5
100	31/5	29	32
150	24/7	22	25
200	16/4	15	17
250	11/2	10	12
300	0	0	0
350	0	0	0
	ES >10 kDa + honey 72h	ES >10 kDa + honey 72h	ES >10 kDa + honey 72h
0	70	70	70
25	36/7	35	37
50	32/1	30	35
100	26/7	25	27
150	19/8	20	21
200	10/94	10	9
250	5/76	4	6
300	0	0	0
350	0	0	0

Promastigote	ES <10 kDa + honey 24h	ES <10 kDa + honey 24h	ES <10 kDa + honey 24h
0	70	70	70
25	56/4	52	54
50	44/92	42	45
100	39/78	36	39
150	32/34	32	33
200	26/7	27	29
250	20/8	21	23
300	11/9	10	12
350	0	0	0
	ES <10 kDa + honey 48h	ES <10 kDa + honey 48h	ES <10 kDa + honey 48h
0	70	70	70
25	48/8	44	49
50	39/3	37	41
100	35/1	32	38
150	27/2	26	29
200	18/6	17	19
250	13/5	12	13
300	6/9	7	10
350	0	0	0
	ES <10 kDa + honey 72h	ES <10 kDa + honey 72h	ES <10 kDa + honey 72h
0	70	70	70
25	42/2	36	45
50	29/6	34	36
100	26/7	32	26
150	19/74	20	24
200	13/9	12	14
250	9/92	9	10
300	0	0	0
350	0	0	0

Promastigote	Honey 24h	Honey 24h	Honey 24h
0	70	70	70
25	55/2	59	52
50	50/3	48	52
100	46/8	49	43
150	40/3	42	38
200	30	34	35
250	25/77	29	27
300	16/85	17	21
350	11/8	10	9
	Honey 48h	Honey 48h	Honey 484h
0	70	70	70
25	49	52	46
50	37/2	42	44
100	35/5	34	39
150	29/1	29	33
200	23/2	24	25
250	17/1	17	20
300	7/36	8	9
350	2	1	3
	Honey 72h	Honey 72h	Honey 724h
0	70	70	70
25	45/6	42	48
50	40/6	36	38
100	30/7	30	31
150	24/1	23	25
200	16/6	16	20
250	10/9	8	9
300	0	0	0
350	0	0	0

Promastigote	Glucantime 24h	Glucantime 24h	Glucantime 24h
0	70	70	70
25	45/66	46	45
50	39/33	38	38
100	31	32	32
150	26/67	27	26
200	20/08	21	22
250	13/66	14	14
300	5/66	6/5	5
350	0	0	0
	Glucantime 48h	Glucantime 48h	Glucantime 48h
0	70	70	70
25	38/35	39	40
50	32/71	32	33
100	25/33	26	26
150	18/33	19	18
200	14/67	15	14
250	6/53	6	5
300	0	0	0
350	0	0	0
	Glucantime 72h	Glucantime 72h	Glucantime 72h
0	70	70	70
25	28	31	31
50	27	26	27
100	20	19	19
150	14	13	13
200	6	5	6
250	2	0	0
300	0	0	0
350	0	0	0

Amastigote			
rate of macrophage infection	Crude ES + honey	Crude ES + honey	Crude ES + honey
150	40	38	42
300	21	18	23
number of amastigotes per infected macrophage cell	Crude ES + honey	Crude ES + honey	Crude ES + honey
150	53	50	57
300	23	26	20

rate of macrophage infection	ES >10 kDa + honey	ES >10 kDa + honey	ES >10 kDa + honey	ES <10 kDa + honey	ES <10 kDa + honey	ES <10 kDa + honey
150	49	47	51	58	61	56
300	44	41	46	49	47	51
number of amastigotes per infected macrophage cell	ES >10 kDa + honey	ES >10 kDa + honey	ES >10 kDa + honey	ES <10 kDa + honey	ES <10 kDa + honey	ES <10 kDa + honey
150	91	89	94	129	133	126
300	67	69	65	103	99	107

rate of macrophage infection	Honey	Honey	Honey	Glucantime 72	Glucantime 72	Glucantime 72
100				39	38	42
150	66	64	69	30	29	32
300	57	56	59			
number of amastigotes per infected macrophage cell	Honey	Honey	Honey	Glucantime 72	Glucantime 72	Glucantime 72
100				78	76	79
150	167	169	165	51	49	53

300	130	127	134			

MTT	Crude ES + honey 48h	Crude ES + honey 48h	ES >10 kDa + honey 48h	ES >10 kDa + honey 48h
25	99	99	99	99
50	99	98	97	98
100	98	97	97	98
150	97	96	97	96
200	97	97/5	96	95
250	96	95	96	95
300	96	96	95	94
350	94	93	94	93/5
	Crude ES + honey 72h	Crude ES + honey 72h	ES >10 kDa + honey 72h	ES >10 kDa + honey 72h
25	97	98	98	97
50	96	97	96	95
100	94	95	94	94/5
150	94	95	93	94
200	91	92	89	90
250	90	91	88	88/5
300	88	87	88	87
350	84	84/5	85	85/5

MTT	ES <10 kDa + honey 48h	ES <10 kDa + honey 48h	Honey 48h	Honey 48h	Glucantime 48h	Glucantime 48h
25	99	99	99/5	99	98	99
50	99	98/5	99	99/5	98	98
100	98	98/2	99	99/3	97	97
150	98	97/5	98	97/5	97	97
200	97	96/5	97	97/2	96	96
250	97	96	97	96/5	94	95
300	95	94/5	97	96/5	94	94
350	95	94	96	96/1	94	94
	ES <10 kDa + honey 72h	ES <10 kDa + honey 72h	Honey72h	Honey 72h	Glucantime 72h	Glucantime 72h
25	98	98/3	99	99/2	97	97
50	95	96	99	98/6	97	96
100	95	95/5	97	96	96	95
150	92	93	97	96	93	92
200	90	91	95	95/4	90	89
250	89	87	94	95	89	88
300	88	87	92	93/1	86	87
350	87	86/7	91	89	86	85

Bradford protein assay			
Concentration	Response1	Response 2	Response 3
1000	1.84	1.8	1.81
500	1.25	1.3	1.299
250	1.01	1.05	1.044
125	0.901	0.909	0.907
62.5	0.825	0.825	0.823
